# Biosynthesis of silver nanoparticles from plant extracts: a comprehensive review focused on anticancer therapy

**DOI:** 10.3389/fphar.2025.1600347

**Published:** 2025-05-14

**Authors:** Pratibha Pandey, Sorabh Lakhanpal, Ashok Kumar Bishoyi, S. Renuka Jyothi, Swati Mishra, Meenakshi Verma, Ajay Singh, Mir Waqas Alam, Safia Obaidur Rab, Mohd Saeed, Fahad Khan

**Affiliations:** ^1^ Centre for Research Impact and Outcome, Chitkara University Institute of Engineering and Technology, Chitkara University, Rajpura, Punjab, India; ^2^ School of Pharmaceutical Sciences, Lovely Professional University, Phagwara, Punjab, India; ^3^ Marwadi University Research Center, Department of Microbiology, Faculty of Science, Marwadi University, Rajkot, Gujarat, India; ^4^ Department of Biotechnology and Genetics, School of Sciences, JAIN (Deemed to be University), Bangalore, Karnataka, India; ^5^ Department of Pharmacology, IMS and SUM Hospital, Siksha ‘O’ Anusandhan (Deemed to be University), Bhubaneswar, Odisha, India; ^6^ University Centre for Research and Development, Chandigarh University, Mohali, Punjab, India; ^7^ School of Applied and Life Sciences, Uttaranchal University, Dehradun, India; ^8^ Department of Physics, College of Science, King Faisal University, Al-Ahsa, Saudi Arabia; ^9^ Department of Clinical Laboratory Sciences, College of Applied Medical Sciences, King Khalid University, Abha, Saudi Arabia; ^10^ Department of Biology, College of Science, University of Hail, Hail, Saudi Arabia; ^11^ Center for Global Health Research, Saveetha Medical College, Saveetha Institute of Medical and Technical Sciences, Chennai, Tamil Nadu, India

**Keywords:** plant extracts, silver nanoparticles, green synthesis, anticancer, nanotechnology

## Abstract

Cancer is a deadly disease and is one of the primary causes of mortality worldwide. Cancer therapy presents significant challenges, such as chemotherapy resistance, high toxicity, recurrence, and metastasis. As a result, the development of novel therapeutic agents for cancer continues to be a top goal to expand the number of efficient treatments available. The advent of nanotechnology is an important turning point in several scientific disciplines. Owing to the increasing difficulty of this problem, researchers have begun to focus their attention on the possibility of employing plants or extracts from plants as a potential tumor treatment. More than 3,000 medicinal plant species have been documented worldwide for their utilization in cancer treatment. Nevertheless, crude plant extracts lack specificity, and their dosages are not clearly specified. To enhance the therapeutic efficacy of these natural substances, researchers have used them in conjunction with silver nanoparticles (AgNPs). Plants possess intricate phytochemical components including sugars, polyphenols, amino acids, flavonoids, terpenoids, alkaloids, and proteins, which can function as reducing and stabilizing agents. In recent years, the application of plant-derived AgNPs has increased significantly, particularly in cancer treatment. These green-synthesized AgNPs are regarded as outstanding tools for the detection of cancer and targeted drug delivery at the tumor site. By leveraging the distinctive characteristics of nanoparticles and the antioxidant and anticancer qualities of plants, these green-synthesized AgNPs selectively eradicate tumor cells while sparing normal healthy cells. This comprehensive review aimed to summarize the key aspects of plant extracts as anticancer agents, biosynthesis of AgNPs, and recent advancements in the antitumor efficacy of green-synthesized AgNPs.

## Introduction

By 2040, the global incidence of cancer is projected to reach 28.4 million, representing a 47% increase from 2020. Cancer is a complex combination of symptoms, characterized by an extensive lack of growth control and persistent progression (Sung et al., 2021; Kah et al., 2023). According to GLOBOCAN, there are an estimated 19.3 million births and 10 million cancer-related instances and fatalities worldwide. The worldwide cancer incidences are projected to reach 28.4 million by 2040 which is 47% increase from 2024. With an anticipated 2.3 million new cases, breast cancer in women has overtaken lung cancer as the most prevalent cancer diagnosis, followed by stomach, colon, lung, and prostate cancers. Additionally, there have been 1.8 million reported deaths, with lung cancer remaining the prominent cause of cancer-related mortality, followed by liver, colorectal, stomach, and breast cancer in women (Deo et al., 2022). As this disease is tissue-based, tissue diversity poses a significant challenge to both diagnosis and therapeutic efficacy (Hassanpour and Dehghani, 2017).

In the last ten years, nanotechnology has been investigated for the development of innovative therapeutic and anticancer drugs (Tran et al., 2020). This is because of the unique features of nanoparticles compared to conventional treatment procedures. Nanomaterials have applications in nanomedicine, particularly in drug delivery and targeted therapy. Additionally, nanomaterials surpass current treatment methods by improving therapeutic efficacy, minimizing toxicity, and exhibiting superior biocompatibility (Padil et al., 2018; Hlapisi et al., 2024). Nanoparticles can be composed of either metals or nonmetals based on their fundamental structures. Metallic nanoparticles are mostly comprised of gold, silver, copper, cobalt, nickel, and semiconductors. Conversely, nonmetallic nanoparticles predominantly consist of carbon-based substances. Extensive investigation has been undertaken on metallic nanoparticles because to their unique optical, electrical, and catalytic characteristics (Bharathi et al., 2018). Silver nanoparticles (AgNPs) have attracted significant interest because of their distinctive features (Dos Santos et al., 2014; Mathur et al., 2018). Silver nanoparticles (AgNPs) are crucial nanomaterials that have been extensively studied owing to their unique biological characteristics.

Silver nanoparticles (AgNPs) have emerged as a compelling and highly adaptable category of nanomaterials, garnering considerable interest from the scientific community in several disciplines (Akter et al., 2017). Silver nanoparticles (AgNPs) can be developed using three methods: physical, chemical, and biological. Plants have proven to be promising options for the production of AgNPs because of their superior scalability, nontoxicity, cost-effectiveness, and straightforward synthesis. Furthermore, their biocompatibility and nonpathogenic properties render them suitable for biomedical applications (Xu et al., 2020). Plants have been used as sources of therapeutic compounds for thousands of years, resulting in the extraction of many pharmaceuticals. Substantial research has been conducted to evaluate the biological activity of plant extracts and their constituents. Plant extracts contain several secondary metabolites such as alkaloids and flavonoids which can function as both capping and reducing agent. Moreover, AgNPs have demonstrated significant potential for oncological research. Their capacity to selectively elicit cytotoxicity in cancer cells while preserving healthy tissue renders them appealing candidates for targeted anticancer therapy (Zielinska et al., 2017). Researchers have thoroughly investigated AgNPs as vehicles for drug delivery, intending to accurately target chemotherapeutic drugs to malignant cells, thus reducing systemic side effects and improving the effectiveness of cancer therapies (Gomes et al., 2021; Xu et al., 2023). The potential of AgNPs to transform cancer therapy is an advancing field of study with significant possibilities for the future of oncology (Jangid et al., 2024). Therefore, this review is meticulously designed to assess the function of AgNPs as plant-derived medication carriers for cancer treatment. Herein, we summarize various *in vitro* studies on plant extract-based AgNPs against several carcinomas. The research articles focused in this review emphasized on *in vitro* investigations of the anticancer properties of plant extract-mediated AgNPs. The underlying mechanism of the antitumor potential of biosynthesized AgNPs has been addressed in certain investigations. The selection process strictly adhered to guidelines for scientific studies published in peer-reviewed journals. The literature review was performed utilizing the following electronic databases: PubMed, Google Scholar, SCOPUS, and Web of Science. The search method was established using the following keywords: plant extract,” “green synthesis,” “biosynthesis,” “silver nanoparticles,” “Ag nanoparticles,” “cancer cell lines,” and “anticancer effect. An identical search strategy was employed across all datasets, with modifications where necessary.

### Efficacy of plant extract as anticancer agent

**FIGURE 1:**
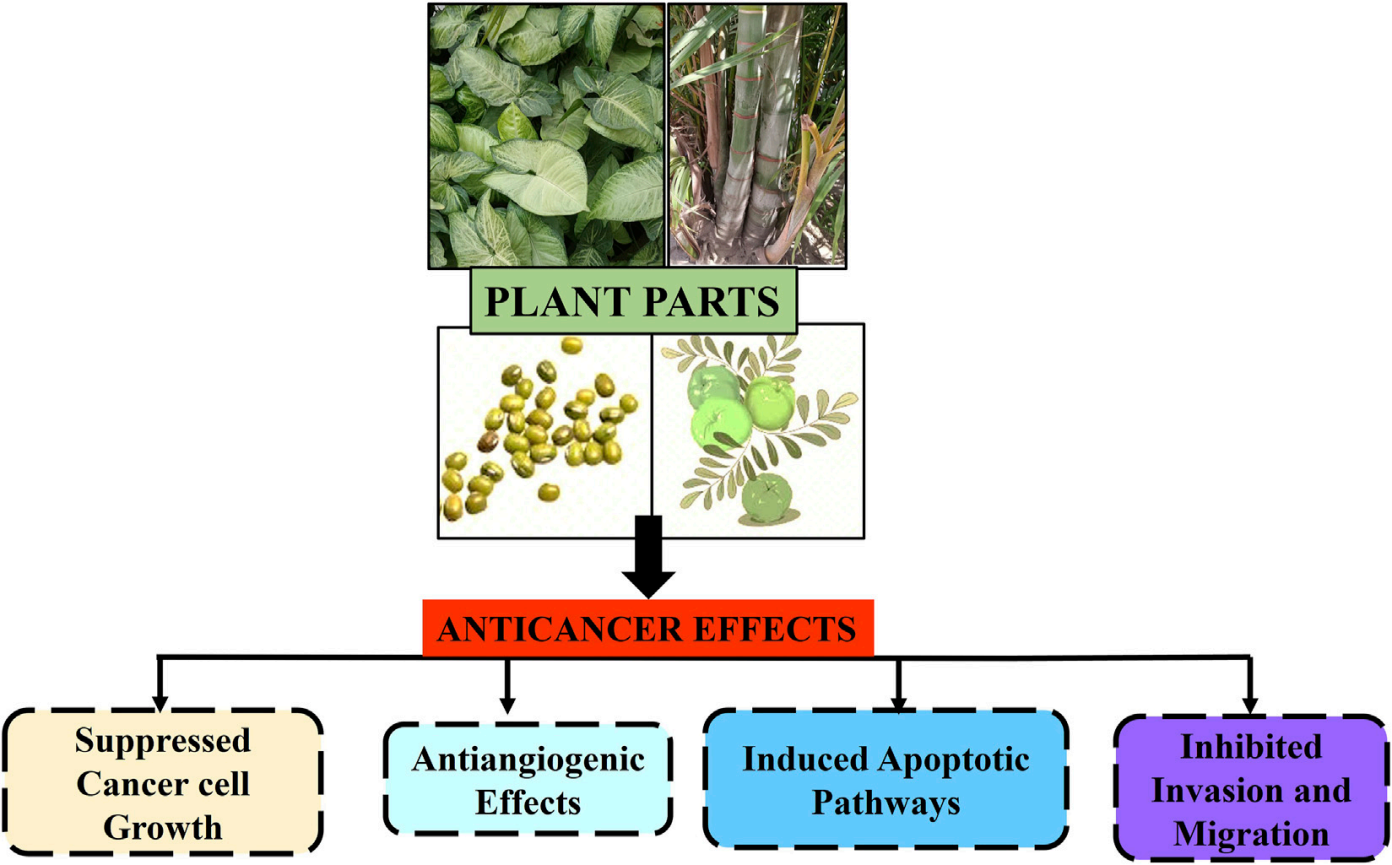
Schematic representation of anticancer effects of plant parts and their extracts against different types of carcinomas.

In Asian and African cultures, medicinal plants have been used as traditional remedies for thousands of years. In Western countries, numerous plants are consumed because of their multiple health advantages. The WHO indicates that certain countries continue to rely on plant-based treatments as their primary form of medicine, while developing nations are harnessing the advantages of naturally derived substances for therapeutic applications (Zahra et al., 2020; Bajpai et al., 2024). Numerous questions have been raised regarding the effectiveness of herbal medicines in enhancing the body’s immune cells against cancer. Consequently, a number of herbal formulations have been developed to fight malignant cells without endangering healthy cells in the body, based on documented information on the intricate synergistic interaction of several phytochemicals with anticancer potential (Craig, 1999). Herbal remedies and medicinal plants play an important role in primary healthcare systems, particularly in rural areas, because conventional anticancer treatments are extremely expensive and out of reach for low-income populations. Herbal remedies have been helpful in cancer prevention and treatment because of their ability to suppress hormones and enzymes that activate cancer, boost the DNA repair mechanism, induce antioxidant action, and improve immunity, all of which contribute to anticancer effects (Greenwell and Rahman, 2015). Several medicinal plants are currently being studied in clinical trials to determine their potential anticancer effects (Figure 1). Researchers have also conducted extensive research on the phytochemicals found in plants (Solowey et al., 2014). Because of the side effects caused by conventional cancer treatments, some patients are turning to complementary and alternative medicine in an attempt to identify a more effective treatment (Gielecińska et al., 2023).

Several studies have utilized traditional material medica literature as a basis for cancer medication development. To develop promising candidates with anticancer properties, it is necessary to thoroughly understand the many phytochemicals found in plants and how they work together (Merina et al., 2012; Shaikh et al., 2016). The capacity to alter one or more particular molecular processes has led to the isolation and identification of several novel chemopreventive drugs. A different and supplementary approach to cancer prevention and/or therapy may be developed as a result of the identification of useful herbs and the clarification of their underlying mechanisms. Several plant-derived anticancer drugs, including vinblastine, camptothecin, and taxol, have been isolated and characterized from different plant species (Cragg and Newman, 2005). Conventional therapeutic options have demonstrated inadequate results in meeting the essential criteria for effective cancer treatment, leading to a significant interest in plant-derived anticancer agents, which are both cost-effective and safe because of their natural nature. Thus, there is potential for the extended use of plant formulations or herbal extracts for anticancer treatment. In recent decades, herbal treatments have been widely adopted, significantly influencing both the global healthcare systems and the international commerce of herbal products. Medicinal plants have been confirmed to play a significant role in the healthcare systems of a substantial portion of the global population (Antonio et al., 2014). Recently, the utilization of plants and plant-derived phytomedicines has surged significantly in developed countries such as the USA owing to their lower toxicity and natural origin. Numerous medicinal plants are traditionally utilized for the treatment and prevention of malignancies worldwide, including India. The promising and rapidly increasing application of plant derived compounds in cancer therapy necessitates comprehensive scientific investigation of diverse and biologically relevant phytochemicals, alongside their detailed anticancer mechanisms and clinical studies, which could represent a compelling area for future cancer research (Shukla and Mehta, 2015).

Despite significant efforts in preclinical studies, the translation of phytochemicals for human use has seen modest success. Poor transport of potential natural agents to the target site is likely the primary reason for clinical failure. This may be attributed to the agents’ inadequate water solubility and chemical instability inside the biological milieu. Moreover, the inadequate pharmacokinetic properties of secondary metabolites of plants, resulting from rapid metabolism, limited solubility, and instability, cause many issues related to toxicity, inefficacy, and diminished tissue distribution. Therefore, it is critical for cancer treatment to find new and improved delivery systems that can overcome these obstacles (Wei et al., 2019).

### Significance of silver nanoparticles

Given the incredible progress that has been made in the field of nanotechnology, it should not come as a surprise that researchers have shown an increasing interest in the topic in recent years. Owing to its intrinsic versatility, nanotechnology is used in a wide range of fields such as electronics, sensors, optics, chemistry, pharmaceuticals, cosmetics, biomedical sciences, and environmental science. There are variations in the characteristics of nanoparticles (NPs) owing to differences in their chemical composition, shape, size, and controlled release. These discrepancies frequently stem from the synthesis process, which is affected by multiple factors. The goal of modern nanoparticle production is to achieve nanoscale dimensions while simultaneously making sure the synthesis method is easy to understand, inexpensive, eco-friendly, and adaptable to many uses (Zhang et al., 2016; Almatroudi, 2020). For the most part, metal nanoparticles, which include silver, gold, and platinum NPs, are rather small, measuring approximately 50 nm, and possess a high surface area. They possess the capability to traverse capillaries within tissues and cells because of their small size. Their enormous surface area, coupled with their capacity for chemical modification, enables them to accommodate a substantial quantity of drugs. In this context, metal nanoparticles have been utilized for the regulated release of drugs in cancer therapy (Patra et al., 2018). Although certain metals are preferred to be inert, bioaccumulation and toxicity may occur. Conversely, a significant benefit of metal nanoparticles is their capacity to effectively absorb light energy and transform it into heat. Consequently, certain agents can be utilized in hyperthermic tumor therapy, wherein photostimulation delivers thermal energy, rendering this treatment very specific (Ahlawat et al., 2020).

Owing to their unique physiochemical and biological intrinsic features, silver nanoparticles (AgNPs) have been used in the pharmaceutical, aerospace, food, and biomedical sectors. They are characterized by high electrical conductivity, biological peculiarities, and excellent optical characteristics (Huy et al., 2020). The AgNPs demonstrated utility in industrial and health-related goods, including the coating of medical equipment, biosensors, bio-imaging, and as antimicrobial, antiparasitic, antiviral, anticancer agents, and drug delivery systems, among various other applications (Shanmuganathan et al., 2019; Sakthi et al., 2022; Kaushal et al., 2023). Owing to their high surface/volume ratio, ease of production, customizable surface chemistry and functionalization, and excellent penetration and traceability throughout the body, silver nanoparticles have been extensively investigated and used in therapies (Marassi et al., 2018). AgNPs are widely used in preclinical studies using different cancer cell models owing to their inherent antitumor potential, but some recent scientific studies have sought to enhance antitumor efficacy by combining AgNPs with conventional anticancer drugs, especially when used in combination with phytocompounds employed in their synthesis (Morais et al., 2020; Gomes et al., 2021). AgNPs offer a novel approach for cancer therapy through nanotechnology as a potential nanoproduct for cancer treatment. Their ability to selectively block the respiratory chain in the mitochondria, which produces reactive oxygen species (ROS) and damages DNA, is one of their proven anticancer properties (Ovais et al., 2016). The production of AgNPs is accomplished by converting silver ions into nanoscale, extremely tiny materials through the application of nanotechnology (Yesilot and Aydin, 2019). Compared to chemical and physical approaches, biogenic (green) methods offer more environmentally friendly ways to convert bulk silver ions into AgNPs. The use of plants in the biogenic synthesis of AgNPs is characterized by cost-effectiveness, reduced risks to humans and the environment, and ease of execution. Nanoscale silver nanoparticles (AgNPs) exhibit novel physicochemical features and can enhance distinctive biological functions (Wei et al., 2015; Kah et al., 2023).

### Mechanism behind anticancer potential

The anticancer capabilities of AgNPs can be explained by a number of processes, as stated in relevant research. It is clear that cancer cells exhibit an enhanced permeation and retention effect (EPR), which causes an influx of AgNPs, which eventually kill cancer cells or at least repress their uncontrolled growth. Furthermore, AgNPs function by modulating signaling pathways, leading to either the induction of early apoptosis or inhibition of rapid tumor cell proliferation. Moreover, certain studies indicate that the stimulation of p53, caspase-3, and p-ErK1/2 by AgNPs ultimately induces apoptosis and modulates cell division through a sequence of intracellular events (Gurunathan et al., 2015; Buttacavoli et al., 2017; Bethu et al., 2018).

Numerous studies have demonstrated that treatment of tumor cells with green-synthesized AgNPs induces ROS, which causes cellular damage (Minai et al., 2013; Ratan et al., 2020). In an experimental finding, researchers ascribe the anticancer effect of green-synthesized AgNPs to the active generation of ROS. Elevated ROS levels affect cell signaling pathways that are crucial for apoptosis activation (Hembram et al., 2018). Existing literature indicates that green-synthesized AgNPs induce ROS production, which eventually results in apoptosis and cell death.

Buttacavoli et al. studied the effect of AgNPs on the SKBR3 breast cancer cell line using advanced methods and procedures. These results provide a definitive understanding of how AgNPs work against human cancer cell lines (SKBR3) to prevent cancer. They documented substantial suppression of cell motility and inhibitory effects on metalloproteinases (MMPs). Notable morphological alterations in cancer cells were observed following treatment with AgNPs, including shrinkage, shape irregularity, cytoplasmic blebbing, alterations in intracellular vacuole morphology, and chromatin condensation. Alongside these alterations in cancer cells, the generation of ROS induces oxidative stress and cellular apoptosis. Additionally, findings documented the elevated levels of LC3-II, ATG7, beclin-1, and ATG5, alongside the decreased levels of HSP90, AKT, P62, and p-AKT autophagic markers (Buttacavoli et al., 2017; Raja et al., 2020). Research indicates that cancer cells exhibit an enhanced permeability and retention (EPR) effect (Maeda, 2010), resulting in the increased uptake of nanosilver and the production of more toxic silver ions. By destroying essential biomolecules, the oxidative stress produced in tumor cells disrupts numerous physiological processes and molecular pathways, leading to the destruction of essential cellular organelles, ultimately resulting in the death of tumor cells (Datkhile et al., 2023; Ullah et al., 2024). The formation of new blood vessels by vascular endothelial growth factor (VEGF) enhances tumor progression and metastasis (Pathak et al., 2024). Studies indicate that AgNPs influence the activity of VEGF, hence functioning as an anti-angiogenic agent (Palanisamy et al., 2024). In addition to reducing the cytotoxic effects of chemotherapy on healthy tissues, silver nanomaterials serve as carrier vehicles for the therapeutic anticancer payload (drugs), increasing the effectiveness and potency of anticancer medications and preventing cancer cells from developing resistance to them (Karuppaiah et al., 2020; Sofi et al., 2022). Lin et al. revealed that AgNPs function as anticancer agents by inducing autophagy in cancer cells via activation of the PI3K pathway. Moreover, they noted that the suppression of autophagy by autophagic inhibitors, such as wortmannin, increased the efficiency of cancer cell eradication in a mouse melanoma cell model (B16 cell lines) (Lin et al., 2014). Jia et al. assessed the impact of AgNPs on human colon cancer HCT116 cells and observed that increasing concentrations of AgNPs diminished cellular activity while elevating intracellular ROS levels. Protein and gene expression analyses demonstrated that AgNPs elevated p38 protein phosphorylation levels and upregulated the expression of Bax and p53. Moreover, Bcl-2 expression is downregulated and the levels of p21 upregulated, which expedites cellular apoptosis (Jia et al., 2020). Nonetheless, green AgNPs may activate some of the aforementioned pathways and potentially other mechanisms because they can be naturally stabilized with bioactive organic molecules during their formation.

### Plants mediated nanoparticles synthesis

**FIGURE 2:**
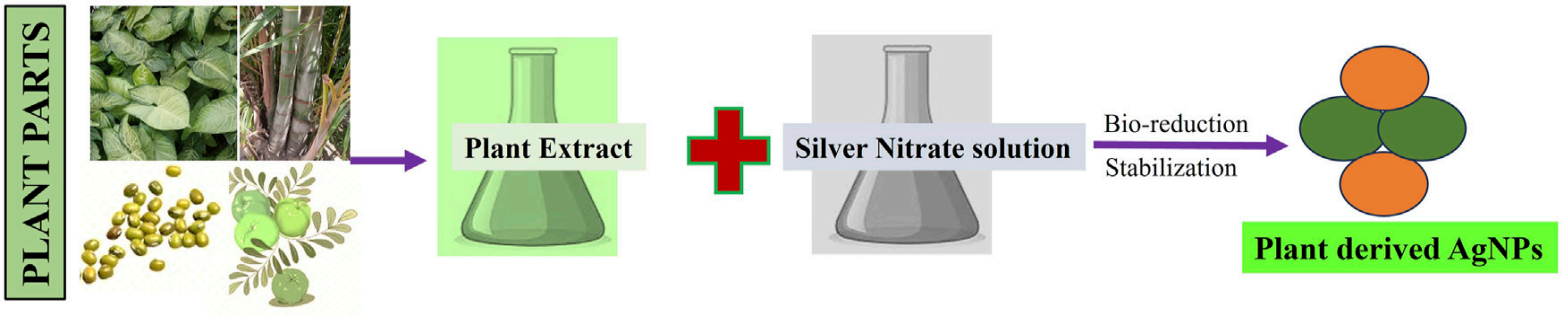
A schematic representation of green synthesis of silver nanoparticle by plant extracts.

To achieve green synthesis of silver nanoparticles (AgNPs), it is possible to make use of live organisms, which represent the kingdom of the biological system. In addition to being essential for the production of food and nutrition, living organisms are also critical for green synthesis. Since many plants have a large biomass, scientists prioritize using them to carry out green synthesis of AgNPs because of their molecular arsenal and biomass richness (Shafey, 2020). The primary and secondary metabolites of plants influence their response to stress factors and survival agents making them principal bioreactors and molecule providers for green synthesis (Jha et al., 2009). Metal ion reduction occurs because of the occurrence of metallic counterparts and the stabilization of the surface of the AgNPs (El-Seedi et al., 2019). Plant derived molecules that contribute to this process include citric acid, amino acids, phenolic compounds, terpenoids, heterocyclic compounds, enzymes, peptides, polysaccharides, saponins, and tannins (Thakkar et al., 2010; Shah et al., 2015; Yosri et al., 2021; Shah et al., 2024). The green synthesis of AgNPs can be achieved by using either the entire plant or its organs or tissues, or extracts from these, along with other plant components (such as seeds, leaves, bark, roots, and fruits) to create nano-objects that exhibit a variety of characteristics (Roy, 2021; Shiraz et al., 2024) (Figure 2).

A plant extract was amalgamated with a metal precursor solution under diverse reaction conditions to synthesize nanoparticles (Malik et al., 2014). Nanoparticle synthesis, productivity, and stability are influenced by the reaction variables regulating the plant extract, including the metal salt quantity, phytochemical quantity, solution temperature, pH level, and phytochemical category (Li et al., 2011). In contrast to bacteria and fungi, which require extended incubation periods, the phytochemicals present in plant extracts have a greater propensity to reduce metal ions within a shorter timeframe. Consequently, plant extracts are regarded as excellent candidates for the biosynthesis of metallic nanoparticles. Plants possess diverse amounts of phytochemicals, which are significant elements in nanoparticle synthesis. Nanoparticle bioreduction mostly involves plant-based natural products such as sugars, carboxylic acids, polyphenols, terpenoids, ketones, and aldehydes (Makarov et al., 2014; Thatyana et al., 2023).

Phytoconstituents of the plant serve as exceptional reducing and stabilizing agents. The flower extract of *Loni cera hypoglauca* functions as a reducing and capping agent in the manufacture of AgNPs and exhibits anticancer action (Jang et al., 2016). Leaf extract of *Artocarpus integer* was utilized to produce silver nanoparticles (AgNPs), resulting in spherical nanoparticles ranging from 5.76 nm to 19.1 nm in size (Majeed et al., 2019). The extract of *Catharanthus roseus* utilized for the synthesis of AgNPs exhibited indole-type alkaloids, which function as reducing and stabilizing agents (Al-Shmgani et al., 2017). Greenly produced silver nanoparticles (AgNPs) utilizing leaf extracts from *Clitoria ternatea* and *Solanum nigrum* have shown antibacterial efficacy against nosocomial infections. The synthesis of nanoparticles was validated by UV, FTIR, SEM, and XRD analyses (Krithiga et al., 2015). The pulp extract of *Abelmoschus esculentus* (L.) was utilized to synthesize AgNPs measuring 3–11 nm, which exhibited anticancer and antibacterial properties (Mollick et al., 2019). In addition to this, several other research studies demonstrate the advanced development of nanoparticles via the green synthesis process and their prospective applications in medicine (Jain et al., 2021).

### Advancement in anticancer effects of plant based AgNPs

**FIGURE 3:**
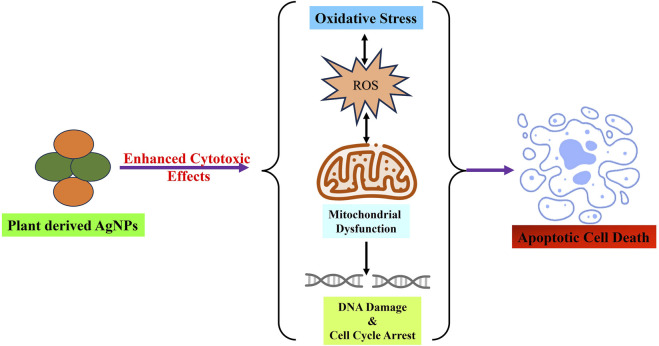
Green synthesized AgNPs from various parts of plant and herbs demonstrated significant cytotoxic potential via inducing oxidative stress, mitochondrial dysfunction, cell cycle arrest and finally leads to apoptotic cell death.

Research on biogenic NPs using plant materials has become an emerging field because of the growing use of NPs in the management and treatment of cancer. Traditional methods of producing nanoparticles (NPs) sometimes require the use of harsh chemical reducing agents, which are not only expensive and complicated, but also have the potential to generate highly harmful byproducts. Consequently, there is an undeniable need for more cost-effective and environmentally benign alternative methods that can mitigate the risk of environmental contamination. This section summarizes the plant-based AgNPs currently utilized in anticancer therapy and examines their use across diverse cancer types (Table 1). Figure 3 depicted the anticancer mode of action of green synthesized AgNPs as therapeutic and cytotoxic agent.

**TABLE 1:** *In vitro* anticancer activity of green synthesized silver nanoparticles from various plant extracts.

Cancer type	Model	Plant	Part used	Dosage and exposure time	Mode of action	Normal cell cytotoxicity	References
Breast cancer	MCF-7 cells	*Alternanthera tenella*	Leaves	25–500 μg/mL; 24 h	Growth inhibitory effects	-	Sathishkumar et al. (2016)
	MCF-7 cells	*Allium cepa*	Leaves	25–50 μg/mL; 24 h	Cytotoxic effects	-	Mani et al. (2021)
	MCF-7 cells	*Aloe barbadensis miller*	Leaves	10–1,000 μg/mL; 24 h	Cytotoxic effects	No	Ghatage et al. (2023)
	T-47D cells	*Camellia sinensis*	Leaves	110–410 μg/mL; 24 h	Antiproliferative effects, G2-M Cell cycle arrest, ROS generation, MMP reduction, Apoptotic induction	No	Tamang et al. (2025)
	MCF-7 cells	*Cassia fistula*	Fruit pulp and leaves	10–100 μg/mL; 48 h	Cytotoxic effects	-	Abaid et al. (2023)
	MDA-MB-231 cells	*Acalypha indica*	Leaves	1, 10, 50 and 100 μg/mL; 48 h	Cytotoxic effects, Apoptotic induction	-	Krishnaraj et al. al. (2014)
	MCF-7 cells	*Citrullus colocynthis*	Fruit	5 μg/mL; 72 h	Reduced proliferation, migration, spheroid size, and colony formation Apoptotic induction	-	Rasool et al. (2022)
	MCF-7 cells	*Brassica oleracea*	Leaves	37 μg/mL; 24 h	Cytotoxic effects, Apoptotic induction	-	Kavipriya et al. (2024)
	MCF-7 cells	*Eucalyptus tereticornis*	Leaves	20–100 μg/mL; 24 h	Cytotoxic effects	-	Kiran et al. (2020)
	MCF-7 cells	*Moringa oleifera*	Leaves	2, 4 and 6 μg/mL; 24 h	Growth inhibitory effects, PI3K/AKT pathway mediated apoptotic induction	No	Permatasari et al. (2025)
	MCF-7 cells	*Moringa oleifera*	Leaves	1.25, 2.5, 5 and 10 μg/mL; 24, 48 and 72 h	Cytotoxic and genotoxic effects	No	Alkan et al. (2022)
	MCF-7 cells	*Phoenix dactylifera*	Fruit	12.5–200 μg/mL; 48 h	Cytotoxic effects via inducing apoptosis, necrosis	-	Zafar and Zafar (2019)
	HCC-712 cells	*Phoenix dactylifera*	Seed	25–500 μg/mL; 24 h	Cytotoxic effects	-	Allemailem et al. (2022)
	MCF-7 cells	*Trigonella foenum-graecum*	Seed	1.56 and 3.12 μg/mL; 24 h	Cytotoxic effects	No	Varghese et al. (2019)
	HCC-712 cells	*Nigella sativa*	Seeds	25–200 μg/mL; 24 h	Cytotoxic effects	-	Almatroudi et al. (2020)
	MCF-7 cells	*Nigella sativa*	Seed	1–100 μg/mL; 24 h	Cytotoxic and apoptotic inducing effects	-	Rohini et al. (2019)
Lung Cancer	A549 cells	*Cannabis sativa*	Seed	0–375 μg/mL; 48 h	Cytotoxic effects	-	Yontar and Çevik (2024)
	A549 cells	*Phoenix dactylifera*	Seed	20–100 μg/mL; -	Cytotoxic effects	-	Khader et al. (2022)
	A549 cells	*Phoenix dactylifera*	Seed	1–100 μg/mL; 24 h	Growth inhibitory effects, Apoptotic induction, ROS generation, MMP reduction, G1 cell cycle arrest	No	Farshori et al. (2022)
	A549 cells	*Mucuna pruriens*	Seed	5–100 μg/mL; 24 h	Cytotoxic effects	-	Menon et al. (2018)
	A549 cells	*Azadirachta indica*, *Gymnema sylvestre*, and *Moringa oleifera*	Leaves	20 μg/mL; 36 h	Antiproliferative and antiangiogenic effects	No	Muthu et al. (2025)
	A549 cells	*Hibiscus sabdariffa*	Leaves	5, 10, and 30 μg/mL; 48 h	Cytotoxic effects	-	Ahirwar et al. (2024)
	A549 cells	*Cymodocea serrulata*	Leaves	10–200 μg/mL; 36 h	Cytotoxic effects	-	Palaniappan et al. (2015)
	A549 cells	*Acorus calamus L. and Dalbergia sissoo Roxb*	Leaves	0.975–2000 μg/mL; 24 h	Growth inhibitory effects, Apoptotic induction, ROS generation, MMP reduction	No	Thakkar et al. (2024)
	A549 cells	*Origanum vulgare*	Leaves	10–500 μg/mL; 36 h	Cytotoxic effects	-	Sankar et al. (2013)
Gastrointestinal cancer	HepG2 cells	*Abutilon hirtum*	Leaves	51 µM; 24 h	Cytotoxic effects	-	Arul and Kothai (2023)
	PANC-1, AsPC-1, and MIA PaCa-2 cells	*Berberis thunbergii*	Leaves	0–1,000 μg/mL; 24 h	Cytotoxic effects	No	Guo et al. (2022)
	Caco-2 cells	*Eucalyptus camaldulensis*	Leaves	5, 10, and 30 μg/mL; 24, 48 and 72 h	Cytotoxic effects	-	Zein et al. (2020)
	HCT116 cells	*Manilkara zapota*	Leaves	2.5–70 μg/mL; 72 h	Growth inhibitory effects, Apoptotic induction, ROS generation, MMP reduction	-	Shaniba et al. (2019)
	HTC116 and SW480 cells	*Moringa oleifera*	Leaves	3.125–100 μg/mL; 48 h	Wnt/β-catenin signaling mediated antiproliferative effects	-	Althomali et al. (2022)
	HT-29 cells	*Moringa oleifera*	Leaves	250–2000 μg/mL; 24 h	Cytotoxic and antimetastatic effects	-	Susanto et al. (2024)
	HepG2 cells	*Nigella sativa*	Seeds	0.5–10 μg/mL; 24 h	Cytotoxic effects	-	Usmani et al. (2019)
	HCT-15 cells	*Vitex negundo*	Leaves	50–250 μg/mL; 24–48 h	Antiproliferative effects, Apoptosis induction, G0/G1 cell cycle arrest	-	Prabhu et al. (2013)
	HT-29 cells	*Zingiber officinale, Curcuma longa*	Rhizome	25–500 μg/mL; 24 h	Cytotoxic effects	-	Venkatadri et al. (2020)
	HT-29 cells	*Curcuma longa*, *Curcuma aromatica*, and *Curcuma caesia*	Rhizome	11.34, 15.45, and 12.67 g/mL; 24 h	Cytotoxic effects	-	Jain et al. (2023)
	AsPC-1, PANC-1, and MIA PaCa-2 cells	*Zingiber officinale*	Rhizome	1–1,000 μg/mL; 24 h	Cytotoxic effects	No	Wang et al. (2021)
	Eca-109 cells	*Photinia glabra*	Leaves	20 ug/mL; 24 h	Cytotoxic effects	-	Namulinda et al. (2023)
Gynecological cancers	HeLa cells	*Euphorbia antiquorum* L.	Fruit	3–50 μg/mL; 48 h	Cytotoxic effects	-	Rajkuberan et al. (2017)
	WI-38 and SK-OV-3 cells	*Hibiscus sabdariffa*	Latex	0.01–100 μg/mL; 48 h	Cytotoxic effects, Apoptotic induction	No	Aboelmaati et al. (2021)
	HeLa cells	*Moringa oleifera*	Stem	15.62–250 μg/mL; 24 h	Growth inhibitory effects, ROS mediated apoptotic induction	-	Vasanth et al. (2014)
Prostate cancer	PC-3 cells	*Dimocarpus longan*	Stem bark	2–20 μg/mL; 72 h	Cytotoxic effects, Apoptotic induction	-	He et al. (2016)
	DU145 cells	*Carica papaya*	Peel	0.5–5.0 μg/mL; 24–48 h	Antiproliferative effects, Cell cycle arrest, Apoptotic induction	No	Singh et al. (2021)
Melanoma	A375 cells	*Moringa oleifera*	Leaves	12.5–25, 50–100, and 200–400 μg/mL; 24 h	Cytotoxic effects	No	Mohammed and Hawar (2022)
Leukemia	THP-1 cells	*Nyctanthes arbortristis*	Leaves	5–50 μg/mL; 24 h	Cytotoxic effects	-	Kumari et al. (2018)
Multiple cancers	MCF-7 and A549 cells	*Jacobaea maritima*	Leaves	0.35–5.5 μg/mL; 24 h	Cytotoxic effects	-	Althubiti et al. (2023)
	MCF7 HeLa, C6 and HT29 cells	*Juglans regia*	Leaves	0–500 μg/mL; 16 h	Cytotoxic effects		Şimşek et al. (2023)
	MCF-7, A549 and Hep-2 cells	*Beta vulgaris*	Root	10–100 μg/mL; 72 h	Growth inhibitory effects Apoptotic induction	-	Venugopal et al. (2017)
	MCF-7 and H1299 cells	*Juglans regia*	Leaves	0.0156–1.0 mg/L; 24 h	Antiproliferative and apoptotic inducing effects	No	Seçme et al. (2024)
	MCF-7, Hep-G2 and HCT-116 cells	*Moringa oleifera*	Leaves	6.51, 4.75, and 5.54 μg/mL; 24 h	Cytotoxic effects	-	Abdel-Rahman et al. (2022)
	MCF-7 and Caco-2 cells	*Moringa oleifera*	Leaves	200–1,200 μg/mL; 48 h	Cytotoxic effects	-	Al Baloushi et al. (2024)
	MCF-7 and HT-29 cells	*Nigella arvensis*	Seed	20–120 μg/mL; 48 h	Cytotoxic effects	-	Chahardoli et al. (2017)
	B16F10 and HepG2 cells	*Parthenium hysterophorus*	Leaves	10–100 μg/mL; 24 h and 48 h	Cytotoxic effects	No	Ahsan et al. (2020)

## Breast cancer

Numerous traditional treatments, such as endocrine therapy, surgery, radiation, and chemotherapy, have been employed for decades to treat breast cancer. Nevertheless, these therapeutic strategies are associated with a range of life-threatening and severe side effects in patients. Moreover, combination therapy is necessary for rapid intervention and to prevent drug resistance to conventional breast cancer treatments. Biogenic AgNPs derived from plants are gaining attention for their use in detecting and treating breast cancer (Akhtar et al., 2024). Sathishkumar et al. synthesized silver nanoparticles (AgNPs) using an aqueous leaf extract of *Alternanthera tenella*, suggesting that flavonoids are responsible for the synthesis of AgNPs. Various imaging techniques, including TEM, ultraviolet spectroscopy, FTIR, and X-ray diffraction, were used to characterize AgNPs. The mean diameter of the nanoparticles was determined to be approximately 48 nm. The anticancer efficacy of AgNPs was evaluated against human breast cancer MCF-7 cells, which revealed a dose-dependent enhancement in cell growth inhibition and a decrease in MCF-7 cell migration. The IC_50_ value of the AgNPs was determined to be 42.5 μg/mL (Sathishkumar et al., 2016). Mani et al. utilized *Allium cepa* leaf extract for the biosynthesis of silver nanoparticles (AgNPs), serving as an effective capping and reducing agent for nanoparticles. The UV–vis spectrum confirmed the production of Ag NPs by displaying a distinctive absorption peak at 438 nm. The crystalline structure of the biosynthesized AgNPs was confirmed by XRD analysis and average size found to be 23 nm. The zeta potential of −19.1 mV derived from DLS investigations signifies the exceptional stability of AgNPs. The MTT assay demonstrates the anticancer efficacy of silver nanoparticles in a dose-dependent manner and is unequivocally a promising agent for breast cancer therapy (Mani et al., 2021).

Ghatage et al. developed silver nanoparticles using an extract of *Aloe barbadensis* miller leaves. The biogenic AgNPs exhibited notable anticancer, antioxidative, antibacterial, and photocatalytic activities. The biosynthesized AgNPs were examined using imaging microscopy, transmission electron microscopy, XRD, ATR-IR, AFM, and FE-SEM. The biogenic AgNPs exhibited a maximum absorption peak at 439 nm and proved their crystalline structure through XRD analysis. The anticancer efficacy of AgNPs was assessed in MCF-7 breast cancer cells using the MTT cytotoxicity test. AgNPs demonstrated anticancer activity at concentrations as low as 10 μg/mL, and with increasing concentrations, their anticancer efficacy increased (Ghatage et al., 2023).

Moreover, Tamang et al. utilized an ethanolic extract of *Camellia sinensis* to develop a straightforward, rapid, cost-effective, and eco-friendly method for the manufacture of silver nanoparticles (AgNPs). Biological assessment of T-47D cells indicated that AgNPs exhibited greater inhibitory efficacy in the T-47D cell line than in the crude extract, without demonstrating any significant inhibitory effect on normal cells. The study indicated that AgNPs induced oxidative stress in breast cancer cells via ROS generation. Moreover, AgNPs induced apoptosis-induced cell death in T-47D cells, as evidenced by the AO/EtBr and Hoechst 33342/PI double staining techniques, in conjunction with the annexin/PI flow cytometry approach. Treatment with AgNPs affects the control of mutant p53 in T-47D cells, resulting in the expression of cell cycle inhibitors (CDKN1A/p21 and Gadd45), which induce cell cycle arrest for repair or death. Flow cytometry study of the cell cycle in T-47D cells showed a statistically significant increase in the G2/M phase of cells following treatment with AgNPs at different doses (Tamang et al., 2025).

Green synthesis of AgNPs was executed out by Abaid et al. using extracts from the fruit pulp and leaves of Cassia fistula. The anticancer effects of the plant extracts and biogenic AgNPs were tested on the breast cancer MCF-7 cells, and the results demonstrated that AgNPs significantly suppressed the growth of tumor cells. This confirms that the AgNPs mediated by C. fistula fruit extract exhibit superior activity compared to their counterparts and may be utilized in biological applications (Abaid et al., 2023). Krishnaraj et al. reported the cytotoxic effects of AgNPs biosynthesized from *Acalypha indica* Linn leaf extract against MDA-MB-231 breast cancer cells. The nanoparticles produced from the plant extract demonstrated considerable cytotoxicity, with apoptotic characteristics verified by stimulation of caspase-3 and DNA fragmentation studies (Krishnaraj et al., 2014). Rasool et al. examined the antitumor efficacy of biosynthesized silver nanoparticles derived from *Citrullus colocynthis* fruit extract. AgNPs had a significant antiproliferative capability against MCF7 cells, as evidenced by suppressed proliferation, migration, and colony formation. Substantial modifications in the expression of cell surface indicators, apoptosis, and cell proliferation markers further validated the antiproliferative effects of AgNPs. In addition, AgNPs demonstrated antilipidemic properties by decreasing cellular cholesterol and triglyceride contents and modulating essential genes associated with lipogenesis (Rasool et al., 2022). Further study indicated the anticancer effectiveness of biosynthesized AgNPs from *Brassica oleracea* leaf extract against MCF-7 breast cancer cells. The UV-visible spectrometer verified that the AgNPs in the sample were synthesized via the reduction of silver ions. Furthermore, the FTIR study yielded more information that corroborated the presence of biological elements in the synthesis procedure. The XRD analysis confirmed the stability and crystalline nature of the nanomaterials, and a particle-size analyzer predicted that the nanomaterials would have a size range of 10–80 nm. The biosynthesized AgNPs exhibited cytotoxic and apoptotic effects on MCF-7 breast cancer cells, with an IC_50_ value of 37.0 μg/mL. The apoptotic potential of AgNPs was additionally validated using AO/EtBr and DAPI labeling techniques (Kavipriya et al., 2024).

According to a study by Kiran et al., *Eucalyptus tereticornis* leaf extract was used as a bioreducing agent to produce AgNPs. The synthesized AgNPs were characterized and validated using several analytical techniques, including UV-Vis spectroscopy, X-ray diffraction (XRD), scanning electron microscopy (SEM) with energy-dispersive X-ray spectroscopy (EDS), and transmission electron microscopy (TEM). XRD analysis was conducted for the crystallographic studies. The size and morphology were analyzed using SEM and TEM, and the elemental composition was verified using EDS. Additionally, the biogenic AgNPs showed significant antitumor potential against breast cancer MCF-7 cells with an IC_50_ of 63.257 μg/ml as assessed by cytotoxic assays (Kiran et al., 2020). Permatasari et al. utilized *Moringa oleifera* Lam. leaf powder to produce biogenic AgNPs. The dye exclusion assay indicated that these AgNPs markedly diminished the viability of breast cancer cells, with no significant cytotoxic effects observed in normal cells after 24 h of examination. AgNPs were found to induce apoptosis via caspase-3-mediated signaling and suppressed levels of AKT phosphorylation (Permatasari et al., 2025).

Alkan et al. demonstrated the anti-breast cancer and genotoxic properties of green-synthesized *Moringa oleifera*-mediated silver nanoparticles (AgNPs). In breast cancer MCF-7 cells, AgNPs were found to have significant cytotoxic effects; however, in normal HUVEC, there was insufficient evidence of toxicity. The IC_50_ value of Moringa oleifera-mediated AgNPs was 5 μg/mL. These AgNPs also induced DNA damage in MCF-7 cell lines (Alkan et al., 2022).

Zafar and Zafar biologically generated silver nanoparticles with an extract from *Phoenix dactylifera* fruits. The biosynthesized AgNPs exhibited substantial cytotoxic potential, with an IC_50_ value of 90.8 μg/mL. These nanoparticles also elicit cytotoxicity through necrosis, apoptosis, and mitodepressive processes that can disrupt cellular components at various phases of the cell cycle (Zafar and Zafar, 2019).

Allemailem et al. showed that the extract of *Phoenix dactylifera* (Ajwa date) can serve as a potential source for the biosynthesis of AgNPs. AgNPs demonstrated significant cytotoxic effects on HCC712 breast cancer cells. With increasing concentrations of AgNPs, the percentage cell viability of breast cancer HCC712 cells declined, and at 100 and 250 μg/mL, considerable cytotoxicity was noted (Allemailem et al., 2022).

Additionally, Varghese et al. employed the seed extract of *Trigonella foenum-graecum* L. for green synthesis of AgNPs. The anticancer efficacy of AgNPs against MCF7 and Vero cells was assessed using the MTT assay. According to the findings, the anticancer ability of AgNPs was found to be directly correlated with the concentration and time periods. These results indicate that MCF7 and Vero cells treated with AgNPs had a high level of cytotoxic activity. The IC_50_ values for these cells were 6.25 and 12.5 μg/mL, respectively (Varghese et al., 2019). Almatroudi et al. evaluated the anticancer efficacy of AgNPs produced using the seed extract of *Nigella sativa* as both stabilizing and reducing agents. AgNPs derived from *Nigella sativa* shown considerable cytotoxic action against human breast HCC-712 carcinoma cells (Almatroudi et al., 2020).

Similarly, Rohini et al. produced AgNPs from an aqueous seed extract of *Nigella sativa*. AgNPs were assessed using a series of tests to determine their effectiveness against human breast cancer MCF-7 cells. The AgNPs produced exhibited dose-dependent cytotoxic effects (1–200 μg/mL) against MCF-7 cells, as indicated by morphological changes. Additionally, the treatment of generated AgNPs modified the expression levels of apoptotic markers, Bax and Bcl-2, as well as inflammatory marker, COX-2, in breast cancer MCF-7 cells (Rohini et al., 2019).

## Lung cancer

Lung cancer remains to pose a significant global health concern, prompting continuous research into novel treatments such as combined and targeted therapies, immunotherapy, and surgical interventions (Megyesfalvi et al., 2023). Biogenic production of AgNPs via plant extracts offers a viable method for biomedical and pharmacological applications. A number of characterisation methods have verified the synthesis and purity of these AgNPs, lending credibility to their potential medicinal uses (Radhakrishnan et al., 2022). Yontar and Çevik synthesized AgNPs using *Cannabis sativa* seed extracts and examined their anticancer activity against A549 lung cancer cells. Fourier transform infrared spectroscopy (FTIR) verified that phenolic groups from plant extracts established novel chemical interactions with AgNPs. AgNPs stabilized with *Cannabis sativa* demonstrated a 100% inhibitory potential at 375 μg/mL against lung cancer A549 cells after 48 h of exposure (Yontar and Çevik, 2024).

Khader et al. established an innovative technique for synthesizing AgNPs from the extract of *Phoenix dactylifera* (Rothan) date seeds, and characterized them using spectral data. This study assessed the antiproliferative properties of AgNPs derived from date seed extracts in the A549 lung cancer cell line. The results demonstrated that these AgNPs exhibited a significant cytotoxic effect on these cells (Khader et al., 2022). Similarly, Farshori et al. produced AgNPs from the extract of *Phoenix dactylifera* (Rothan) date seeds and assessed their anticancer efficacy against lung cancer cells. The AgNPs exhibited concentration-dependent antiproliferative effects in A549 cells, with an IC_50_ value of 9.27 μg/mL. The cytotoxicity of AgNPs was linked to ROS generation and depolarization of the mitochondrial membrane potential. Additionally, flow cytometry revealed a concentration-dependent rise in the G0/G1 population and late apoptotic cells, confirming AgNPs-induced apoptosis in A549 cells (Farshori et al., 2022). Menon et al. synthesized AgNPs using *Mucuna pruriens* seed extract. The biosynthesized nanoparticles were characterized using UV-Vis spectroscopy, FTIR, SEM, and energy dispersive X-ray spectroscopy (EDAX). AgNPs suppressed the growth of lung cancer A549 cells in a dose-responsive manner, as evaluated by MTT assay. The IC_50_ value was documented as 50 μg in comparison to the standard drug (Menon et al., 2018).

Muthu et al. produced silver nanoparticles (AgNPs) with plant extracts from *Gymnema sylvestre*, *Moringa oleifera*, and *Azadirachta indica* to assess their anticancer efficacy, specifically their impact on gene expression in A549 lung cancer cells. AgNPs demonstrated significant cytotoxicity against A549 cells, showing greater efficiency than the leaf extracts alone. Gene expression analysis revealed a notable decrease in VEGF and CYCLIN-D1, indicating that there may be repressive effects on angiogenesis and cell cycle progression (Muthu et al., 2025).

Ahirwar et al. investigated the anticancer properties of AgNPs synthesized from *Hibiscus sabdariffa* extracts. AgNPs showed cytotoxicity against the human lung cancer A549 cell line and exhibited anti-inflammatory properties (Ahirwar et al., 2024). Palaniappan et al. developed silver nanoparticles (AgNPs) with the aqueous extract of the seagrass *Cymodocea serrulata* and assessed their cytotoxic effects on lung cancer cells. The colloidal AgNPs have demonstrated remarkable *in vitro* anticancer efficacy against human lung cancer A549 cell lines, with an LD_50_ value of 100 μg/mL (Palaniappan et al., 2015).

Additionally, Thakkar et al. documented the chemical and environmentally friendly manufacture of AgNPs using extracts from *Acorus calamus* L. and *Dalbergia sissoo* Roxb. The anticancer efficacy of the manufactured nanoparticles was evaluated using an MTT assay in human lung cancer A549 and normal lung WI-38 cells. The IC_50_ values for *Dalbergia sissoo* NPs *and Acorus calamus* NPs were 14.25 ± 1.85 μg/mL and 21.75 ± 0.498 μg/mL, respectively, against A549 cells. Further, in addition to preventing cancer cell invasion, the nanoparticles produced by NPs altered mitochondrial membrane potential (MMP), produced reactive oxygen species (ROS), and showed the ability to trigger apoptosis (Thakkar et al., 2024). Sankar et al. synthesized AgNPs using an aqueous leaf extract of *Origanum vulgare*. The experimental results indicated that 50% cell mortality occurred in A549 cells at a dose of 100 μg/mL. Researchers have shown that bioactive substances, such as thymol, linalool, quercetin, apigenin, sabinene, carvacrol, terpinolene, and terpinene, in *Origanum vulgare* enhance the cytotoxic action of AgNPs by functioning as capping agents (Sankar et al., 2013).

### Gastrointestinal cancers

Despite the prevalence of gastrointestinal cancer, the existing diagnostic and treatment methods are insufficient (Zhao et al., 2020). Many metallic nanoparticles (MNPs), particularly AgNPs, have been extensively studied for various medicinal uses, including cancer. They have the ability to overcome the difficulties posed by traditional chemotherapy and have a major effect on the overall survival of patients with gastrointestinal cancer. AgNPs with targeted ligands enhanced the localization of tumor energy deposition, improved solubility and stability, and exhibited specific targeting characteristics. In recent years, numerous researchers have demonstrated and elucidated the cytotoxicity of AgNPs, developed via green technologies, in different gastrointestinal cancer cell lines (Roshani et al., 2023). This section presents an overview of green-manufactured AgNPs and their advancements in targeted gastrointestinal cancer therapy in preclinical research.

Arul and Kothai produced AgNPs using the whole plant extract of *Abutilon hirtum* (Lamp), which were evaluated for their cytotoxic efficacy against HepG2 cell lines. The MTT experiment demonstrated that the produced AgNPs had a substantial cytotoxic effect on HepG2 cell lines (Arul and Kothai, 2023). Guo et al. synthesized AgNPs using *Berberis thunbergii* leaf extract, demonstrating great selectivity and increased cytotoxicity against three different pancreatic cancer cell lines (PANC-1, AsPC-1, and MIA PaCa-2). Furthermore, the IC_50_ values of the AgNPs were determined to be 259, 268, and 141 μg/mL when tested against the PANC-1, AsPC-1, and MIA PaCa-2 cell lines specifically (Guo et al., 2022). Likewise, the extract of *Eucalyptus camaldulensis* leaves has been employed for the biosynthesis of AgNPs. This study examined the anticancer efficacy of colloidal silver nanoparticles derived from *Eucalyptus camaldulensis* leaves against human colon cancer Caco-2 cells. The cytotoxicity experiment on the Caco-2 cell line demonstrated both a dose- and time-dependent effect of AgNPs. After 48 h of exposure, a low concentration (5 μg/mL) decreased cell viability to 50%. Exploring new avenues for treating human colon cancer Caco-2 could be facilitated by the strong cytotoxicity of AgNPs at low concentrations (Zein et al., 2020).

Shaniba et al. produced AgNPs using leaf extracts from *Manilkara zapota* (L.). This study examined the cytotoxic effects of biosynthesized nanoparticles on HCT116 colon cancer cells. After 72 h of treatment, AgNPs dose-dependently and selectively suppressed the development of colorectal cancer HCT116 cells, with an IC_50_ value of 8 μg/mL, while having no effect on the growth of normal cell lines. Using scanning electron microscopy and fluorescence, AgNP-mediated induction of apoptosis was confirmed. The treatment of cells with AgNPs in conjunction with cisplatin resulted in the overproduction of ROS, a decrease in mitochondrial membrane potential, overexpression of apoptotic-related genes including PUMA, caspases, and BAX, and an increase in PARP cleavage (Shaniba et al., 2019). AgNPs were biosynthesized using the leaf extract of *Moringa oleifera*. The cytotoxicity of these biogenic AgNPs was assessed using the MTT assay in colon cancer cells (HTC116 and SW480). Biogenic AgNPs enhanced the cytotoxic effects in a dose-dependent manner as the dose increased from 3.12 to 100 μg/mL. The IC_50_ value for *M. oleifera*-AgNP against HTC116 cells was 70 μg/mL, but that for SW480 cells was 100 μg/mL. *M. oleifera* AgNPs reduced the expression of the CTNNB1 and LRP6 genes, although the expression of the LRP5 gene increased in both cell lines. Following treatment, APC gene expression was diminished in SW480 cells and augmented in HTC116 cells. Overall findings suggest that AgNPs produced by *M. oleifera* extract may effectively prevent colon cancer progression by regulating Wnt/β-catenin signaling (Althomali et al., 2022). Likewise, Susanto et al. demonstrated the antiproliferative efficacy of AgNPs biosynthesized from *Moringa oleifera* in HT-29 cells. MTT assay results indicated that AgNPs produced from *Moringa oleifera* leaf extract significantly reduced HT-29 cell viability and proliferation in a dose-dependent manner. AgNPs also decreased the expression of cell cycle- and proliferation-associated genes including Ki-67, Wnt, β-catenin, and Cyclin D1. Moreover, biogenic AgNPs decreased the expression of metastasis-associated genes, including TGF-β and Snail, recognized as inducers of metastasis (Susanto et al., 2024).

Usmani et al. manufactured AgNPs from the seed extract of *Nigella sativa* and evaluated their efficiency against hepatocellular carcinoma using HepG2 cell lines. Morphological changes and cell viability experiments in HepG2 cells indicated that *N. sativa* AgNPs efficiently limited cell growth in a dose-dependent manner. It has been demonstrated that *N. sativa* AgNPs have considerable anticancer potential against human hepatocellular carcinoma, as evidenced by the disintegration of apoptotic nuclei and the resulting increase in ROS generation. This was accomplished without any effect on normal cells (Usmani et al., 2019). Moreover, AgNPs biosynthesized from the leaf extract of *Vitex negundo* reduced proliferation and caused apoptosis in HCT15 colon cancer cells. AgNPs have substantial antiproliferative effects on human colon cancer cells *in vitro* because of their weak chemical interactions with leaf extract components. According to these findings, AgNPs induced apoptosis by causing G0/G1 cell cycle arrest. Cells treated with AgNPs exhibited DNA damage and increased oxidative stress indicators, which accounted for the cytotoxic effects (Prabhu et al., 2013). Venkatadri et al. have simultaneously employed aqueous extracts of the rhizomes of *Zingiber officinale* and *Curcuma longa* for the green production of silver nanoparticles (AgNPs). This study examined the cytotoxic effects of biosynthesized AgNPs on HT-29 colon cancer cells. Based on the MTT experiment, AgNPs exhibited strong anticancer action against HT29 cells, with an IC_50_ value of 150.8 μg/mL (Venkatadri et al., 2020). In a similar manner, Jain et al. developed silver nanoparticles utilizing various species of the rhizome of *Curcuma longa*. All nanoparticles derived from the rhizome extracts repressed the viability of HT-29 cells to varying degrees. HT-29 cells exhibited sensitivity to the formulated nanoparticles, which elicited increased cytotoxicity against cancer cells (Jain et al., 2023). Additionally, silver nanoparticles were synthesized using the aqueous leaf extract of *Zingiber officinale*. The cytotoxicity of AgNPs was evaluated using the MTT assay on pancreatic cancer cells. Biogenic silver nanoparticles exhibited significant anti-pancreatic cancer effects against PANC-1, AsPC-1, and MIA PaCa-2 cells, while demonstrating minimal cytotoxic action towards normal cell lines (Wang et al., 2021). The synthesis of AgNPs using a green approach utilizing *Photinia glabra* fruit extract has been documented. The antiproliferative effects of the synthesized AgNPs on Eca-109 cells were evaluated. The experimental data indicate that PG-AgNPs exhibited significant cytotoxicity against esophageal cancer cells, with IC_50_ values below 20 μg/mL (Namulinda et al., 2023).

### Gynecological cancers

Unregulated cell proliferation in female reproductive organs is the hallmark of gynecological cancers, which can be characterized as cervical, ovarian, vaginal, uterine, and vulvar malignancies. These malignancies pose considerable risks to women’s health, affecting their lifespans, quality of life, and fertility. More than 116,000 new cases of gynecological malignancies were reported in 2024 (Keyvani et al., 2022). Gynecological malignancies represent a significant worldwide health concern for women and are characterized by elevated incidence rates and an urgent need for enhanced prognostic outcomes (Suneja and Viswanathan, 2020). Current therapeutic techniques include surgical intervention, chemotherapy, radiation, and immunotherapy. Although surgery is mostly useful for solid tumors in their early stages, there is a chance that the tumor will not be completely removed and that it may spread or be implanted (Chen et al., 2019). Chemotherapy is associated with cytotoxicity and reduced bioavailability, constraining its extensive use (Akbarali et al., 2022). There is a limited therapeutic applicability for immunotherapy, and it is not appropriate to tumor forms that have immune suppression or exclusion-like properties (Tan et al., 2020; Zhou et al., 2025).

Recently, nanomedicine has made it possible to create tailored cancer treatments, addressing the primary challenge of existing systemic treatment methods. Combination therapy mediated by nanoparticles can be utilized in conjunction with efflux pump inhibitors, pro-apoptotic substances, and MDR-targeted siRNA (Mignani et al., 2015). AgNPs exhibit potential cytotoxicity in cancer cells and have demonstrated significant anticancer efficacy (Sriram et al., 2010). AgNPs can trigger apoptosis and autophagy, two essential processes in the breakdown of different tumor cell types (Zhang and Gurunathan, 2016). Inhibition of autophagy amplifies the anticancer efficacy of AgNPs (Lin et al., 2014). Additionally, AgNPs can cause apoptosis and suppress the proliferation and survival of cells in numerous cancer cell lines, such as breast, cervical, lung, ovarian, and prostate cancer (Gurunathan et al., 2015; Yuan and Gurunathan, 2017; Yuan et al., 2017; Darabdhara et al., 2019). As a result, using nanoparticles to treat gynecological cancers is a cutting-edge treatment with enormous promise. Rajkuberan et al. demonstrated the quick synthesis of silver nanoparticles using aqueous latex extract of *Euphorbia antiquorum* L to assess its anticancer effectiveness in the cervical cancer HeLa cell line. Phyto-fabricated AgNPs have demonstrated considerable antitumor efficacy against human cervical carcinoma cells (HeLa). Microscopical and preliminary biochemical investigations showed that biosynthesized AgNPs cause significant cytotoxicity in HeLa cells with an IC_50_ value of 28 μg (Rajkuberan et al., 2017). Aboelmaati et al. conducted another work that generated biogenic and biocompatible silver nanoparticles (AgNPs) from the non-edible stem of *Hibiscus sabdariffa*. This study examined the antitumor efficacy of biogenic AgNPs against the ovarian cancer cell lines WI-38 and SK-OV-3. Biogenic AgNPs demonstrated apoptotic and anti-ovarian cancer effects with an IC50 value six times greater than that of the normal cell line (Aboelmaati et al., 2021).

Vasanth et al. synthesized green silver nanoparticles (AgNPs) using *Moringa oleifera* stem bark extract. Electron and atomic force microscopy images were obtained to examine the surface morphology of the AgNPs. The impact of the manufactured AgNPs was assessed in human cervical cancer cells (HeLa), and cell morphology was analyzed using nuclear staining dye, which showed remarkable cytotoxic effects. The efficacy of green produced AgNPs was examined using fluorescence activated cell sorting (FACS) and demonstrated to trigger apoptosis via reactive oxygen species (ROS) production in HeLa cells (Vasanth et al., 2014).

## Prostate cancer

Prostate cancer is a predominant cause of mortality in males globally, especially in the USA and European countries, with over 1.9 million new cases and over 580,000 deaths per year, as per the latest global figures. The management of prostate cancer poses considerable clinical difficulties because of the disease’s pronounced metastatic capability, particularly to essential organs, including the lungs, liver, bones, and brain (Posdzich et al., 2023). Prostate cancer cells are inherently heterogeneous with a wide range of genomic, molecular, and phenotypic characteristics. This makes traditional therapy approaches more difficult, highlighting the urgency for improved diagnostic and therapeutic approaches (Saadh et al., 2025).

Consequently, plant-mediated production of nanoparticles is the most optimal method for targeted delivery to cancer cells while mitigating undesirable toxicity. Medicinal herbs provide a prevalent kind of alternative therapy. Numerous medicinal plants have been utilized in addition to traditional treatment methods (Gurib-Fakim, 2006). Nanoparticles generated from plant extracts or herbal products are being considered as a potential chemopreventive therapy for various types of cancer (Ahmed et al., 2019). As a result of advancements in environmentally friendly synthesis techniques, metal nanoparticles, specifically silver nanoparticles (AgNPs), are gaining significance as therapeutic and diagnostic tools for malignancies. He et al. emphasized the potential of silver nanoparticles (AgNPs) produced from the aqueous extract of *Dimocarpus longan* Lour peel as prospective anticancer agents for prostate cancer. AgNPs, evaluated using several analytical techniques, exhibit significant cytotoxicity against human prostate cancer (PC-3) cells. The trypan blue experiment validated their anticancer efficacy, with IC_50_ value between 5 and 10 μg/mL. AgNPs exhibited dose-dependent cytotoxicity towards PC-3 cells by reducing stat 3, bcl-2, and survivin levels, while simultaneously increasing caspase-3 activity (He et al., 2016).

Similarly, the *Carica papaya* leaf extract-derived silver nanoparticles (AgNPs) showed strong anticancer properties, especially against the prostate cancer cell line DU145. Treatment of DU145 cells with AgNPs (0.5–5.0 μg/mL) for 24–48 h resulted in a reduction in the total cell count by 24%–36%. Suppression of cell growth was correlated with cell cycle arrest at the G2/M phase at 24 h, along with G1 and G2/M phase arrest at 48 h. AgNPs augmented the generation of ROS and played a role in apoptosis induction in DU145 cells. The stimulation of apoptosis (57%) was demonstrated in DU145 cells using AO/EB staining, accompanied by the activation of crucial apoptotic protein markers (Bax, cleaved caspase-3, and cleaved PARP). In addition to increasing the levels of tumor suppressor proteins p21 and p27, AgNPs decreased the cell cycle marker cyclin D1 (Singh et al., 2021).

### Melanoma

Malignant Melanoma, the most fatal variant of skin cancer, is an aggressive and resilient tumor that originates from melanocytes (Castro-Pérez et al., 2023). Melanoma has become a major global public health problem in recent years due to its constantly rising prevalence (Janda and Sinclair, 2022). Recent research has shown that the use of specific nutrient-rich food ingredients can prevent their spread. Although conventional therapies may be applicable in the initial phases of this condition, metastatic melanoma persists as an uncontrolled disease owing to its capacity to evade drug-induced apoptosis (Pellegrini et al., 2021; DeWane et al., 2022). Recently, treatments based on nanomaterials have garnered considerable attention. Silver nanoparticles (AgNPs) possess unique physicochemical and biological properties, leading to diverse biomedical applications (Islam et al., 2021; Naganthran et al., 2022). AgNPs are mostly produced using physicochemical techniques. These conventional methods are generally hazardous, unstable, expensive, lengthy, and detrimental to biodiversity. The manufacturing of nanoparticles must be biocompatible, economically viable, and environmentally friendly for application in the pharmaceutical sector (Ashique et al., 2022). Among the biological methods for synthesizing AgNPs, the synthesis of plant extracts is notably favored owing to its enhancement of herbal therapeutic properties, facilitation of the prolonged release of active constituents, and reduction of the necessary dosage. The biosynthesis of AgNPs presents a promising avenue mostly utilized for antibacterial and anticancer therapies (Simon et al., 2022).

Mohammed and Hawar examined the anticancer efficacy of biosynthesized silver nanoparticles derived from *Moringa oleifera* leaf extract. The cytotoxicity test results indicated that the AgNPs exhibited significant activity against the proliferation of the A375 cell line in comparison to WRL68 normal cells. Varying doses of AgNPs assessed the cell viability rate, demonstrating the capacity of AgNPs to inhibit cancer cell proliferation in a dose-dependent manner. The nanoparticles exhibited minimal efficacy against the A375 cell line at concentrations of 12.5 and 25 μg/mL, yielding effectiveness rates of 73.23% and 62.58%, respectively, with an IC_50_ of 31.65 (Mohammed and Hawar, 2022).

### Leukemia

Despite significant advancements in leukemia therapy and successful chemotherapy outcomes, patient survival remains limited, making access to viable alternative treatments for this malignancy essential. Global efforts are now underway to develop new technologies that can successfully eliminate tumors without seriously harming healthy cells, thereby overcoming the fundamental drawbacks of chemotherapy (Short et al., 2020). Accordingly, nanotechnology has emerged as one of the most promising cancer treatment solutions over the past 10 years. Metal nanoparticles in cell cultures and human tissues result in elevated levels of inflammatory products, including cytokines, and heightened oxidative stress, ultimately culminating in cell death. Nanoparticles are typically assimilated by the nucleus and mitochondria, resulting in DNA alterations, mitochondrial structural damage, and potential cell death (Jurj et al., 2017; Liu et al., 2020). Green nanoparticle production, characterization, and applications have recently emerged as major players in the subfield of nanotechnology. Xie et al. explored the green production of AgNPs using an aqueous extract of *Nyctanthes arbortristis*. In its current form, this synthetic approach is easy to use, inexpensive, and reproducible. To evaluate the formation of silver nanoparticles (AgNPs), various techniques such as X-ray diffraction, DLC, SEM, and UV-visible spectroscopy were utilized. The phytochemicals primarily responsible for nanotransformation were flavonoids, phenols, and glycosides found in the leaves. The *in vitro* cytotoxicity of AgNPs was assessed against human leukemia THP-1 cells at varying doses (5–50 μg/mL). Cell viability was reduced in THP-1 cells treated with AgNPs at concentrations ranging from 5 to 50 μg/mL. The findings indicates that the half maximum inhibitory concentration (IC_50_) is 33.5 μg/mL (Kumari et al., 2018).

## Multiple cancers

The distinctive characteristics of nanoparticles (NPs) have attracted the interest of researchers across multiple disciplines (Salem and Fouda, 2021). Several investigations have highlighted the unique characteristics of nanoparticles, including low melting points, optical and magnetic attributes, large surface-area-to-volume ratios, and outstanding durability. These properties make nanoparticles useful in biomedical fields such as diagnostics, medicine, drug delivery, and the agricultural sector (Siddiqi et al., 2018; Nicolae-Maranciuc et al., 2022; Vorobyova et al., 2022). Silver nanoparticles (AgNPs) have garnered attention as nanomaterials owing to their distinctive physicochemical and biological features (El-Sherbiny and Sedki, 2019; Ahmad et al., 2024). The biological manufacturing of nanoparticles has numerous advantages, such as diminished generation of hazardous by-products, enhanced stability, and lowered toxicity to healthy cells. Moreover, biogenic synthesis is cost-effective and facilitates the rapid production of efficient nanoparticles (Nikolova et al., 2023). Plant extracts, fungi, bacteria, and algae have been suggested as safe sources for the production of nanoparticles in previous studies. In contrast to chemical methods, green approaches facilitate the development of nanoparticles with diverse morphology, each possessing distinct properties (Bhagat et al., 2023). Plants serve as an efficient and rapid source of diverse nanoparticles (Karnwal et al., 2024). AgNPs using *Jacobaea maritima* leaf extract were produced by Althubiti et al. This leaf is well known for its therapeutic effects and functions as a stabilizing and reducing agent. Biosynthesized AgNPs were shown to have a dose-dependent cytotoxic effect on MCF-7 and A-549 cells at concentrations of 0.35–5.5 μg/mL following a 24-h exposure period. At the same quantities of AgNPs that were exposed to both cell types, the leaf extract did not show any cytotoxic effects. The proliferation of MCF-7 and A-549 cells decreased, and a suppressive effect was noted at a dose of approximately 1.4 μg/mL. Over 80% cellular inhibition was observed at the maximum dose of 5.5 μg/mL, in contrast to 1.4 μg/mL. The anticancer investigation of AgNPs demonstrated an IC50 of 1.37 μg/mL for MCF-7 and 1.98 μg/mL for A549 cells (Althubiti et al., 2023).

Şimşek et al. elucidated the anticancer properties of silver nanoparticles synthesized using green methods utilizing aqueous extract from *Juglans regia* (walnut) leaves. Cytotoxicity results indicated that AgNPs possess cellular inhibitory effects on malignant MCF7 (breast), HT29 (colorectal), HeLa (cervix), and C6 (glioma) cell lines (Şimşek et al., 2023). Venugopal et al. produced silver nanoparticles (AgNPs) utilizing beetroot (*Beta vulgaris*) extract as the reducing agent. In comparison to the plant extract, biogenic AgNPs showed significant antitumor activity against breast cancer cells (MCF-7), lung cancer cells (A549), and pharyngeal cancer cells (Hep-2). In comparison to the Beta vulgaris extract, cell viability decreased as the dose of the test Ag-NPs increased. This could be because AgNPs stimulate ROS and interact with biological components, causing cell death. The results of the AO/EtBr staining showed that apoptosis in cancer cells was the mechanism by which Ag-NPs caused cell death (Venugopal et al., 2017).

Seçme et al. produced silver nanoparticles (AgNPs) utilizing walnut leaves and examined their antiproliferative effects on L929, MCF-7, and H1299 cell lines. Characterization studies indicated that AgNPs were well-formed, and cellular investigations demonstrated that the synthesized Ag NPs possess significant anticancer properties. The MTT cytotoxicity test was utilized to assess the antiproliferative activity of AgNPs derived from walnut leaf extract in breast cancer (MCF-7) and lung cancer (H1299) cells. AgNPs exhibited no harmful effects on the mouse fibroblast cell line (L929) utilized as a control but showed significant efficacy against MCF-7 and H1299 cells. Investigations on the apoptosis and necrosis index were conducted through cell counting in randomly selected locations, revealing that these silver nanoparticles decreased cell viability by triggering apoptosis in selected cancer cells (Seçme et al., 2024).

Abdel-Rahman et al. conducted the biogenic production of silver nanoparticles (AgNPs) using a straightforward and eco-friendly process that utilizes an aqueous extract of *Moringa oleifera*. The synthesized AgNPs demonstrated significant cytotoxic effects on colon cancer (HCT-116), hepatocellular cancer (HepG-2), and breast cancer (MCF-7) cells (Abdel-Rahman et al., 2022). Similarly, Al Baloushi et al. synthesized AgNPs using an aqueous extract of *Moringa peregrina* leaves. The antitumor efficacy of the nanoparticles was examined against MCF-7 and Caco-2 cancer cell lines. The findings indicated that the anticancer efficacy of the nanoparticles against MCF-7 and Caco-2 demonstrated that the silver nanoparticles exhibited considerable cytotoxic effects on the examined cancer cell lines, with IC_50_ values of 41.59 μg/mL for Caco-2 and 26.93 μg/mL for MCF-7 (Al Baloushi et al., 2024). Chahardoli et al. utilized an extract of *Nigella arvensis* L. seed powder to create AgNPs from a solution of silver nitrate (AgNO_3_). Green AgNPs were assessed for potential antitumor cytotoxicity against human MCF-7 and HT-29 cell lines using the MTT assay. These biologically generated nanoparticles had significant growth inhibitory effects against breast cancer (MCF-7) and colorectal cancer (HT-29) cells (Chahardoli et al., 2017). Ahsan et al. produced AgNPs using a green chemistry method derived from a *Parthenium hysterophorus* leaf extract. The cell survival rate significantly decreased to less than 70% for AgNPs against B16F10 cells and less than 65% for HepG2 cells after 24 h, even at lower concentrations (5 μg/mL), indicating the effective cytotoxicity of biogenic AgNPs. At elevated concentrations of AgNPs, cytotoxicity was markedly increased, resulting in a decrease in cell viability to approximately 12.54% for HepG2 cells and 17.24% for B16F10 cells after 24 h. The IC50 values for the synthesized nanoparticles against the B16F10 and HepG2 cancer cell lines were 10.18 μg/mL and 10.531 μg/mL for 24 h, and 8.016 μg/mL and 9.87 μg/mL for 48 h, respectively (Ahsan et al., 2020).

### Limitations and future perspectives

The application of plant-mediated synthesized AgNPs as prospective anticancer drugs is promising; however, numerous hurdles and future considerations must be addressed. Prior to human testing, AgNPs must be thoroughly evaluated for their toxicity, biocompatibility, and adverse effects (Stensberg et al., 2011; Levard et al., 2012). Over the past 10 years, a number of researchers have experimentally demonstrated that AgNPs are harmless *in vitro*, which differs greatly from *in vivo* settings (Hadrup et al., 2018; Upputuri and Pramanik, 2020). The toxicological properties of AgNPs are significantly influenced by parameters such as their size, morphology, surface curvature, and concentration (Ferdous and Nemmar, 2020). Important investigations have shown that silver and other nanoparticles are both acutely and chronically harmful *in vivo* (Cunningham et al., 2021). Although there is a widespread perception of the safety of medicinal plants and their extracts, it is crucial to recognize the presence of harmful medicinal plants when not administered at the appropriate dosage (Mugale et al., 2024). Therefore, extensive toxicity studies are essential to assess the safety of nanoparticles generated from medicinal plants for therapeutic applications. Although numerous studies have examined the efficacy of nanoparticles manufactured from medicinal plants, there has been comparatively little emphasis on their selectivity and safety in non-target cells. Long-term toxicity studies are essential to understand the cumulative effects of extended exposure, examine potential chronic toxicity, and evaluate the persistence of nanoparticles in the body over time (Ovais et al., 2016).

A further concern is the assurance of biocompatibility of the green-synthesized AgNPs. Although these nanoparticles demonstrate medicinal properties, their relationship with biological systems must be thoroughly studied to prevent toxicity or adverse reactions. The precise and effective administration of nanoparticles to designated cells or tissues continues to pose a problem. Formulating methods to precisely target cancer cells while reducing off-target effects is essential for efficient cancer treatment. AgNPs may aggregate or become unstable under physiological conditions. Maintaining stability and minimizing undesirable aggregation during preservation and administration are crucial for ensuring effectiveness. Optimization of the therapeutic application of NPs requires a comprehensive understanding of their pharmacokinetics and biodistribution. Their effectiveness and dispersion inside the body are influenced by various factors, including size, surface characteristics, and clearance mechanisms (Asam Raza et al., 2023). Furthermore, a number of additional recent studies have shown that nanoparticles are gradually excreted in urine and feces; nevertheless, the precise mechanism and important metabolic pathways involved remain unclear and require further investigation. The prolonged accumulation of nanoparticles in essential organs might result in significant toxicity; therefore, biodegradability and clearance concerns must be resolved by comprehensive mechanistic investigations prior to initiating clinical trials (Ovais et al., 2016; Drobne et al., 2018).

## Conclusion

Biological methods, particularly green synthesis utilizing plant extracts, offer environmentally sustainable, economical, and biocompatible options. These approaches have been emphasized for their ability to efficiently generate bioactive nanoparticles. Optimization is necessary to improve scalability and industrial usability. Toxicity concerns persist as a significant challenge, considering their extensive potential use in biomedical science and therapies. Research has highlighted the necessity of area-specific criteria and consistent toxicity evaluation techniques. Green synthesis techniques are particularly advantageous for mitigating toxicity. In light of the escalating risk of cancer-related mortality, AgNPs are emerging as viable alternatives to traditional chemotherapy drugs, demonstrating their potential use in cancer treatment and management. The literature predominantly emphasizes the assessment of the anticancer effects of green-synthesized AgNPs. Additional *in vitro*, *in vivo*, and *ex vivo* studies are necessary for preclinical and clinical evaluation of green-produced AgNPs for early diagnosis and treatment of cancer. These therapeutic approaches may soon replace the chemically generated AgNPs in cancer theranostic applications. A great deal of work is required to advance the field of green synthesis of biogenic AgNPs for use in cancer therapeutics. Furthermore, to develop viable anticancer agents, it is necessary to conduct in-depth research on various concerns, including bioavailability, biocompatibility, toxic effects, and clearance of AgNPs. Comprehensive dialogues concerning the sustainability of plant-based AgNPs foster a greater understanding of nanoparticle chemists committed to advancing this objective and ensuring an environmentally friendly future for AgNPs in research, industry, commerce, and cancer treatment.
